# An innovative model based on N7-methylguanosine-related lncRNAs for forecasting prognosis and tumor immune landscape in bladder cancer

**DOI:** 10.1186/s12935-023-02933-7

**Published:** 2023-05-08

**Authors:** Lei Ren, Xu Yang, Jinwen Liu, Weifeng Wang, Zixiong Liu, Qingyuan Lin, Bin Huang, Jincheng Pan, Xiaopeng Mao

**Affiliations:** 1 Department of Urology, The First Affiliated Hospital of Sun Yat-Sen University, Sun Yat-Sen University, No.58 Zhongshan 2nd Road, Guangzhou, 510080 Guangdong China; 2 Department of Urology, The Seventh Affiliated Hospital of Sun Yat-Sen University, Sun Yat-Sen University, Shenzhen, China

**Keywords:** Bladder cancer (BCa), N7-methylguanosine, lncRNA, Prognostic model, Tumor microenvironment, Immunotherapy

## Abstract

**Background:**

As a novel type of the prevalent post-transcriptional modifications, N7-methylguanosine (m7G) modification is essential in the tumorigenesis, progression, and invasion of many cancers, including bladder cancer (BCa). However, the integrated roles of m7G-related lncRNAs in BCa remain undiscovered. This study aims to develop a prognostic model based on the m7G-related lncRNAs and explore its predictive value of the prognosis and anti-cancer treatment sensitivity.

**Methods:**

We obtained RNA-seq data and corresponding clinicopathological information from the TCGA database and collected m7G-related genes from previous studies and GSEA. Based on LASSO and Cox regression analysis, we developed a m7G prognostic model. The Kaplan–Meier (K-M) survival analysis and ROC curves were performed to evaluate the predictive power of the model. Gene set enrichment analysis (GSEA) was conducted to explore the molecular mechanisms behind apparent discrepancies between the low- and high-risk groups. We also investigated immune cell infiltration, TIDE score, TMB, the sensitivity of common chemotherapy drugs, and the response to immunotherapy between the two risk groups. Finally, we validated the expression levels of these ten m7G-related lncRNAs in BCa cell lines by qRT-PCR.

**Results:**

We developed a m7G prognostic model (risk score) composed of 10 m7G-related lncRNAs that are significantly associated with the OS of BCa patients. The K-M survival curves revealed that the high-risk group patients had significantly worse OS than those in the low-risk group. The Cox regression analysis confirmed that the risk score was a significant independent prognostic factor for BCa patients. We found that the high-risk group had higher the immune scores and immune cell infiltration. Furthermore, the results of the sensitivity of common anti-BCa drugs showed that the high-risk group was more sensitive to neoadjuvant cisplatin-based chemotherapy and anti-PD1 immunotherapy. Finally, qRT-PCR revealed that AC006058.1, AC073133.2, LINC00677, and LINC01338 were significantly downregulated in BCa cell lines, while the expression levels of AC124312.2 and AL158209.1 were significantly upregulated in BCa cell lines compared with normal cell lines.

**Conclusion:**

The m7G prognostic model can be applied to accurately predict the prognosis and provide robust directions for clinicians to develop better individual-based and precise treatment strategies for BCa patients.

**Supplementary Information:**

The online version contains supplementary material available at 10.1186/s12935-023-02933-7.

## Background

Bladder cancer (BCa), one of the most prevalent malignancies worldwide with high mortality and morbidity, accounts for around 550,000 new cases and 200,000 fatalities annually [1]. Based on its histomorphological characteristic, BCa can be divided into non-muscle-invasive BCa (NMIBC) and muscle-invasive BCa (MIBC), with occurrence rates of 75% and 25%, respectively [2]. However, approximately 60% of NMIBC patients will suffer a recurrence within 1 year after transurethral resection of BCa, and up to 17% will ultimately develop into MIBC or distant metastases [3]. Once it progresses to MIBC, these patients will have low 5-year survival, below 50% [4]. Over the past few years, there have been remarkable advancements in anti-cancer drugs for BCa, including cisplatin-based combination, intravesical treatment, and immunotherapy, which have improved the prognosis of BCa patients. Nevertheless, the effectiveness of these treatment regimes in some patients is still far from satisfactory due to the high heterogeneity of tumors [5, 6]. Therefore, developing a robust model for evaluating the prognosis and effectiveness of chemotherapy and immunotherapy drugs is essential to provide individual-based treatment and ameliorate the outcomes for BCa patients.

N7-methylguanosine (m7G) is a novel type of the prevalent post-transcriptional modification of RNA, where the seventh N of guanine (G) in RNA is attached to a methyl group. m7G modification functions in modulating gene expressions and presents in mRNA 5’ cap, rRNA, tRNA, and miRNA to regulate the processing, function, and metabolism of RNA for active participation in biological and pathological processes [7, 8]. In humans, the complex of the methyltransferase like 1/WD repeat domain 4 (METTL1/WDR4) catalyzes m7G of tRNA under the action of methyltransferases, which could regulate the mRNA translation [9, 10]. A large body of evidence indicated that based on the abnormal expressions of m7G, this complex plays a fundamental role in the initiation, progression, and invasion of numerous malignancies, including teratoma, colon cancer, liver cancer, lung cancer, as well as BCa [11]. Ying et al. found that METTL1 was high expressed in BCa tissues, and its expression level was significantly correlated with poor outcome of BCa patients; they further demonstrated that m7G tRNA modifications mediated by METTL1 could modulate the translation of EGFE/EFEMP1 and promote the progression of BCa [12]. Moreover, the expression levels of METTL1 and WDR4 were also significantly upregulated in lung cancer tissues, and METTL1-mediated m7G tRNA modifications can regulate the mRNA translation to promote the proliferation and invasion of lung cancer, revealing m7G modification as a key oncogenic factor [13]. In addition, further proof was gathering to suggest that m7G tRNA modifications regulated by the METTL1/WDR4 complex had critical roles in the occurrence, proliferation, and invasions of many cancers, such as intrahepatic cholangiocarcinoma [14], HCC [15], and nasopharyngeal carcinoma [16]. These studies suggest that m7G modifications significantly correlate with tumorigenesis, progression, invasion, and metastasis.

Long non-coding RNAs (lncRNAs) are a major class of non-coding RNA (ncRNA) transcripts with a length greater than 200 nucleotides but are unable to encode proteins or peptides [17]. They are involved in a wide range of cellular activities, including the cell cycle, differentiation and metabolism, post-transcriptional control, as well as regulation of cell signaling [17–20]. It is now well recognized that lncRNAs also have an essential effect on cancer cell proliferation, migration, and invasion in a variety of tumors [20–23]. For example, lncRNA LNMAT1 could promote BCa-related lymphatic metastasis and lymphangiogenesis via CCL2 dependent macrophage recruitment [24]. Additionally, lncRNA DLEU2 was significantly upregulated in gastric cancer and could promote gastric cancer cell proliferation, invasion, and migration [25]. A growing body of evidence has revealed that modifications (m6A, m1A, m5C and others) of lncRNAs play an important role in either cancer promotion or tumor suppression. For instance, m6A lncRNA MALAT1 could regulate metastasis to control cancer cell proliferation, migration, and apoptosis in many cancers; lncRNA XIST bearing m6A and m5C modifications may act as a tumor suppressor or oncogene in colorectal cancer and leukemia [26, 27]. Recently, Wang et al. identified two vital m7G modified lncRNA, which were significantly associated with hypoxia pulmonary hypertension [28]. These suggested that m7G modification presents in lncRNAs, and m7G modified lncRNAs may play a vital role in the progression of many diseases, including cancers. However, the mechanisms and prognosis functions of m7G-related lncRNAs in BCa have yet to be clarified.

In this study, we performed an in-depth bioinformatic analyses to develop an innovative prognostic model comprising ten m7G-related lncRNAs. Then, we assessed its predictive capacity of prognosis, immune checkpoint gene expression levels, tumor mutation burden (TMB), and tumor immune microenvironment (TME). We also investigated whether this model was excellent in predicting chemotherapy sensitivity and immunotherapy response for BCa patients. Finally, we validated the expression levels of these ten m7G-related lncRNAs in BCa cell lines by qRT-PCR.

## Methods and materials

### Public data collection and processing

Original RNA-seq transcriptome data and clinicopathological information of 376 BCa patients were downloaded from the TCGA database (https://tcga-data.nci.nih.gov/tgca/), including 358 BCa samples and 18 normal tissues. For use in our analyses, we extracted RNA-seq data in the fragment per kilobase million (FPKM) format and count data. We eliminated patients lacking clinicopathological data and overall survival time (OS) of fewer than 30 days in an effort to minimize bias as much as feasible. With the “caret” R package, we randomly separated all BCa patients into two sets in a 1:1 ratio: the training and the test.

We screened m7G-related genes from previous literature [29] and Gene Set Enrichment Analysis (GSEA, http://www.gsea-msigdb.org/gsea/index.jsp) with the three gene sets, including GOBP_7_METHYLGUANOSINE_RNA_CAPPING, GOMF_M7G_5_PPPN_DIPHOSPHATASE_ACTIVITY, and GOMF_RNA_CAP_BINDING. Then, we obtained a total of 143 m7G-related genes after removing duplicates (Additional file 1: Table S1). The Pearson correlation analysis evaluated the relationship between lncRNAs and the m7G-related genes. With the criteria of |coefficient R^2^|> 0.4 and p < 0.001, we gained 3201 m7G-related lncRNAs. Moreover, we identified 508 m7G-related lncRNAs that were differentially expressed between normal samples and BCa samples with the standards of p. adj. < 0.05 and |log_2_Fold Change (FC)|≥ 1 using the “Deseq2” R package for the next bioinformatics analyses [30].

### Differentially expressed m7G-related genes and functional enrichment analysis

Differentially expressed m7G-related genes were identified based on the criterion of p. adj. < 0.05 and |log_2_FC|≥ 1 using the “Deseq2” package in R. Then, we conducted KEGG and GO functional analyses to identify related biological processes and enriched signaling pathways using the “clusterProfiler” and “enrichplot” R packages with statistical significance of a false discovery rate (FDR) < 0.25 and p. adj. < 0.05.

### Development and validation of m7G-related lncRNA prognostic model

Based on the survival time in clinicopathological data provided by the TCGA database, we identified 24 m7G-related lncRNAs significantly linked with the OS (p < 0.05) through univariate Cox regression analysis among 508 differentially expressed m7G-related lncRNAs, and the forest plot was created. To reduce the possibility of overfitting, we conducted LASSO regression in the training set for further analyses with 1,000 cycles using the “survival” and “glmnet” R packages. Finally, we ascertained ten optimal lncRNAs and developed an m7G-related lncRNA prognostic model (m7G prognostic model) by multivariate Cox regression. The risk scores for each patient were calculated using the formula: Risk Score = $\sum_{i=1}^{n}(coefi\left(lncRNAi\right)*\mathrm{exp}(lncRNAi))$. Where exp represents the normalized expression values of each lncRNA, and coefi is the regression coefficient determined by multivariate Cox regression. We sorted out all BCa patients into the low-risk and high-risk groups using the median risk score as a cut-off. Kaplan–Meier (K-M) survival analysis was performed to compare the OS between the two risk groups using the “survival” R package. Then, the receiver operating characteristic (ROC) curves measured by the area under the curve (AUC) values were used to evaluate the predictive accuracy of the m7G prognostic model by the “ROC”, “survival”, and “rms” R packages [31]. Besides, we utilized principal component analysis (PCA) to evaluate the capacity of our model to discriminate BCa patients in our study into the two risk groups. Univariate and multivariate Cox regression analyses were conducted to determine whether the risk score was an independent OS prognostic indicator in BCa patients among clinicopathologic features. The results were shown in the forest plots.

### Nomogram and calibration curves

A nomogram combining clinicopathologic features (gender, age, TNM stage, and tumor stage) and the risk score was constructed to improve the clinical application of our model in predicting the OS for BCa patients by the “rms” and “survival” R packages. Utilizing the calibration curve and concordance index (C-index), we determined whether the predicted OS by the nomogram coincided with the actual OS at 1, 3, and 5 years. Besides, time-dependent ROC curve was utilized to assess the efficacy of the nomogram in predicting prognosis for BCa patients with the “timeROC” package in R.

### Functional enrichment analysis

Gene set enrichment analysis (GSEA) was conducted to analyze potentially enriched pathways of the two groups using GSEA software (version 4.3.2, http://www.gsea-msigdb.org/gsea/index.jsp) [32]. P. adj. < 0.05 and simulated value = 1,000 were considered statistically significant. Furthermore, we explored differentially expressed genes (DEGs) between the high- and low-risk groups through the “Deseq2” R package with the criterion of p. adj. < 0.05 and |log_2_FC|≥ 1. Then, we applied KEGG and GO functional analyses to identify related molecular mechanisms and differentially enriched signaling pathways using the “clusterProfiler” and “enrichplot” packages in R [33]. The biological process (BP), cellular component (CC), and molecular function (MF) make up the three components of GO.

### Analysis of tumor immune microenvironment

Tumor immune microenvironment (TME), where tumor cell proliferation occurs, has attracted considerable attention as an emerging therapeutic target. Therefore, we explored immune cell infiltration, immune cell levels, and proportions of immune and stromal cells components in TME. Firstly, ssGSEA algorithm was performed to determine how the subsets of immune infiltrating cells differed between the two risk groups. Then, the immune cell levels of the two risk groups were evaluated using CIBERSORT algorithm. Meanwhile, the correlations between immune infiltrating cells and the risk score were investigated using Pearson correlation analysis (p < 0.05). Based on the “estimate” package in R, the ESTIMATE algorithm was applied to calculate the immune, stromal, and estimate scores (immune scores + stromal scores) to compare proportion of immune and stromal cell components of the TME between the two risk groups. Finally, differentially expressed immune checkpoint genes were compared in the two risk groups, and the result was presented in a box plot.

### Prediction of drug sensitivity

Based on the “pRRophetic” R package, we predicted the IC50 values of the widely recognized anti-BCa drugs for the low- and high-risk groups on the Genomics of Drug Sensitivity in Cancer (GDSC; https://www.cancerrxgene.org/) database. To evaluate the response and outcome of immunotherapy of BCa patients, we extracted the tumor immune dysfunction and exclusion (TIDE) scoring from the TIDE website (http://tide.dfci.harvard.edu) and calculated the TIDE score, immune dysfunction score, and immune exclusion score. Then, we investigated the underlying differences in immune checkpoint blockade (ICB) responses between the two risk groups using the “ggpubr” R package. In addition, we also predicted the response to anti-PD1 and anti-CTLA4 immunotherapy in the two risk groups by the “submap” R package.

### Tumor mutation burden

We obtained the somatic mutation data of BCa patients from the TCGA database. Then, we analyzed and assessed TMB between the low- and high-risk groups using the “maftools” package in R. Based on the median value of TMB, patients in the TCGA set were divided into the low- and high-TMB groups, and the differences in the OS between the two TMB groups were investigated by K-M survival analysis. Combining the risk score and TMB, we divided all patients into four subgroups, and K-M survival analysis was conducted. Furthermore, we calculated correlations between the risk score and TMB by Pearson correlation analysis.

### Cell culture

Human bladder cancer cells T24, UMUC3, and J82 and human normal bladder epithelium cell line SVHUC-1 were purchased from Procell (Procell Life Science & Technology Co., Ltd). Cells were cultured in RPMI-1640 medium (Invitrogen) mixed with 10% FBS. The incubator was set in a water-saturated atmosphere with 5% CO2 at 37 °C.

### Quantitative real-time PCR (qRT-PCR)

The TRIzol (Invitrogen, USA) reagent was utilized to extract total cellular RNA based on the protocol. RNA was reverse transcribed to cDNA by the PrimeScript RT reagent kit (EZBioscience, China). EZBioscience 2 × SYBR Green qPCR Master Mix (EZBioscience, China) conducted the procedure. Primers for qRT-PCR were provided by TSINGKE (Beijing TSINGKE Biotech Co., Ltd., China) and shown in Additional file 2: Table S2. ACTB was chosen for internal reference. Expression levels of lncRNAs were measured as 2-ΔΔCT.

### Statistical analysis

R software (ver.4.1.3) was used to analyze data and visualize the results. Pearson correlation analysis was applied to calculate the correlation coefficient between variables. The log-rank test was performed to determine statistically significant differences between K-M survival curves. SPSS version 25 (IBM SPSS Statistics for Windows, version 25.0, Inc., Chicago, IL, USA) was used to perform the univariate and multivariate Cox regression analysis to confirm the independent prognostic risk factor of the risk score. Statistical significance was defined as p-value < 0.05 when no special description exists.

## Results

### Identification of differentially expressed m7G-related lncRNAs in bladder cancer

**Fig. 1:**
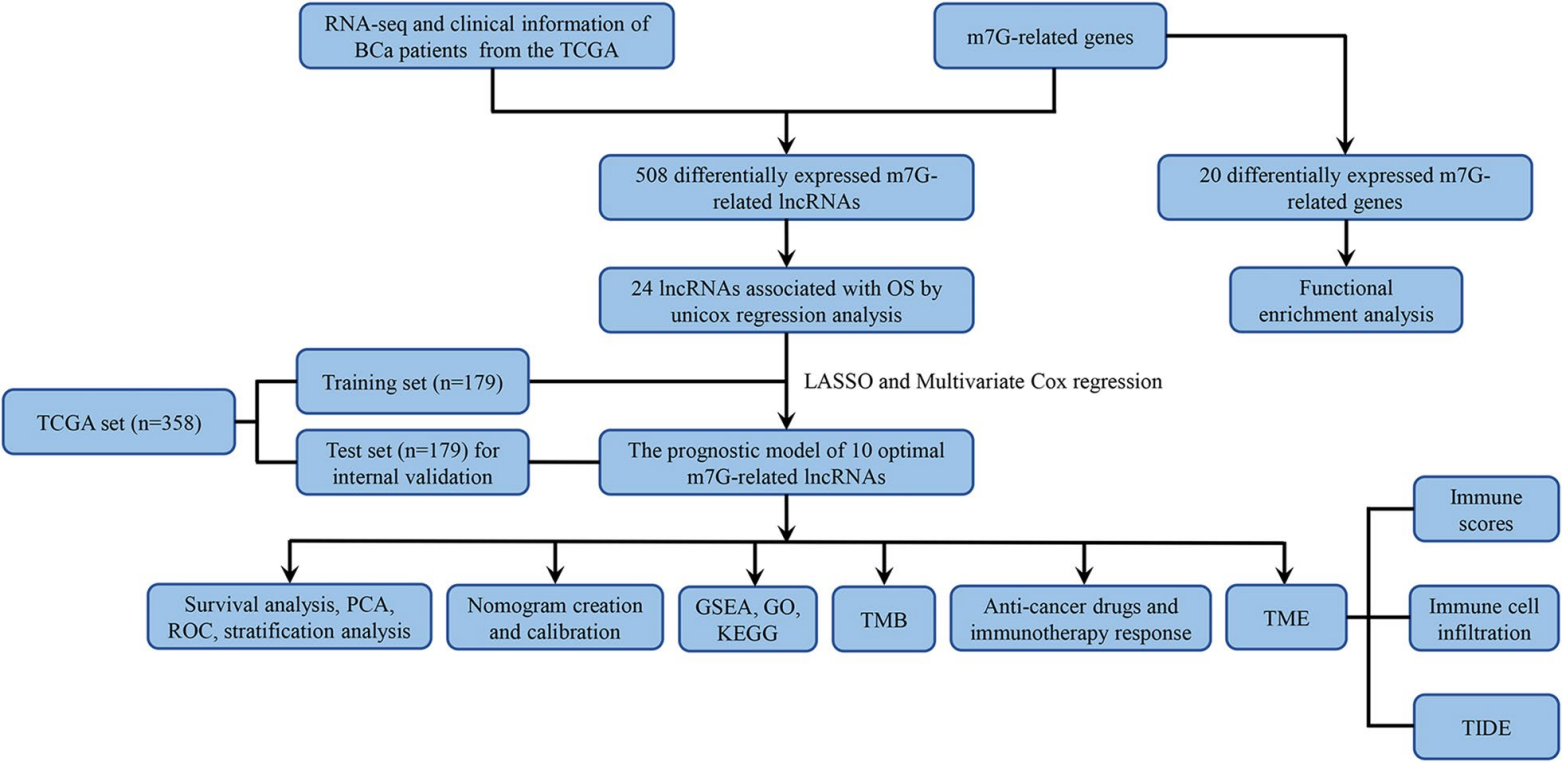
The flow chart of this study

**Fig. 2:**
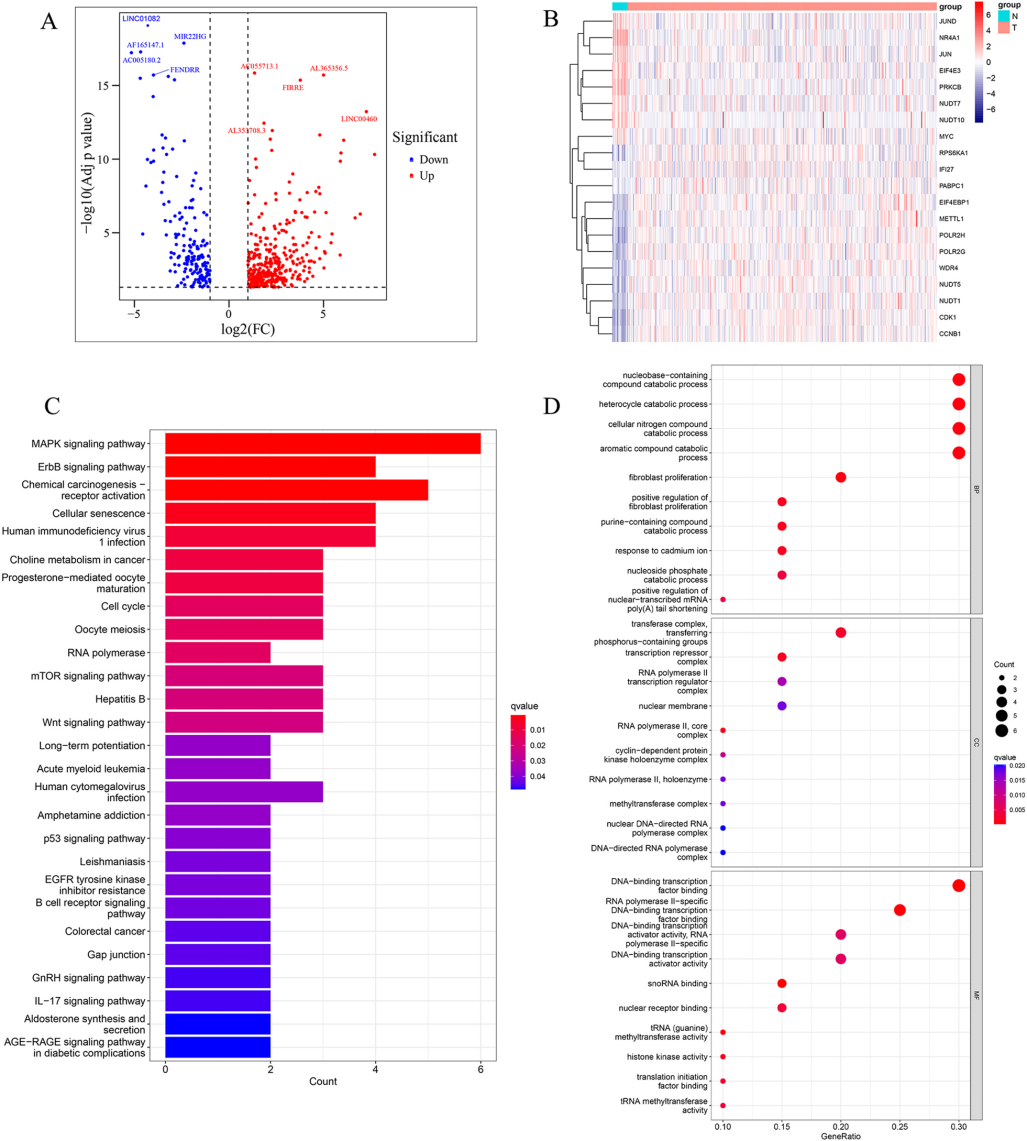
Volcano plot of m7G-related lncRNAs and functional enrichment analysis of differentially expressed m7G-related genes.** A** The volcano plot of 508 differentially expressed m7G-related lncRNAs. **B** The heatmap of differentially expressed m7G-related genes between normal and BCa samples. **C-D** KEGG and GO enrichment analysis of differentially expressed m7G-related genes

Figure 1 illustrates the flow chart of our study. Firstly, after removing duplicates, we collected 143 m7G-related genes from the previous studies and GSEA. According to their expression levels and Pearson correlation analysis, we got 3201 m7G-related lncRNAs with the |coefficient|> 0.4 and p < 0.001. Finally, a total of 508 differentially expressed m7G-related lncRNAs between normal samples and BCa samples (|log_2_FC|≥ 1 and p. adj. < 0.05) were obtained, among which 172 lncRNAs were downregulated and 336 lncRNAs upregulated (Fig. 2A). To investigate the potential mechanism of m7G-related genes in BCa, we identified 20 m7G-related genes that were differentially expressed between normal samples and BCa samples and conducted functional enrichment analysis (Fig. 2B). The KEGG analysis indicated that these genes were mainly enriched in the MAPK signaling pathway, ErbB signaling pathway, chemical carcinogenesis receptor activation, mTOR signaling pathway, and Wnt signaling pathway (Fig. 2C). In regards to the biological process (BP) of GO analysis, these genes were mainly enriched in transcription from positive regulation of nuclear-transcribed of mRNA poly(A) tail shortening, heterocycle catabolic process, and positive regulation of fibroblast proliferation. In regards to the cellular component (CC), these genes were mainly enriched in cyclin-dependent protein kinase holoenzyme complex, methyltransferase complex, and RNA polymerase II transcription regulator complex. In regards to the molecular function (MF), these genes were mainly enriched DNA-binding transcription factor binding, snoRNA binding, and tRNA (guanine)-methyltransferase activity (Fig. 2D).

### Establishment and validation of the m7G prognostic model

**Fig. 3:**
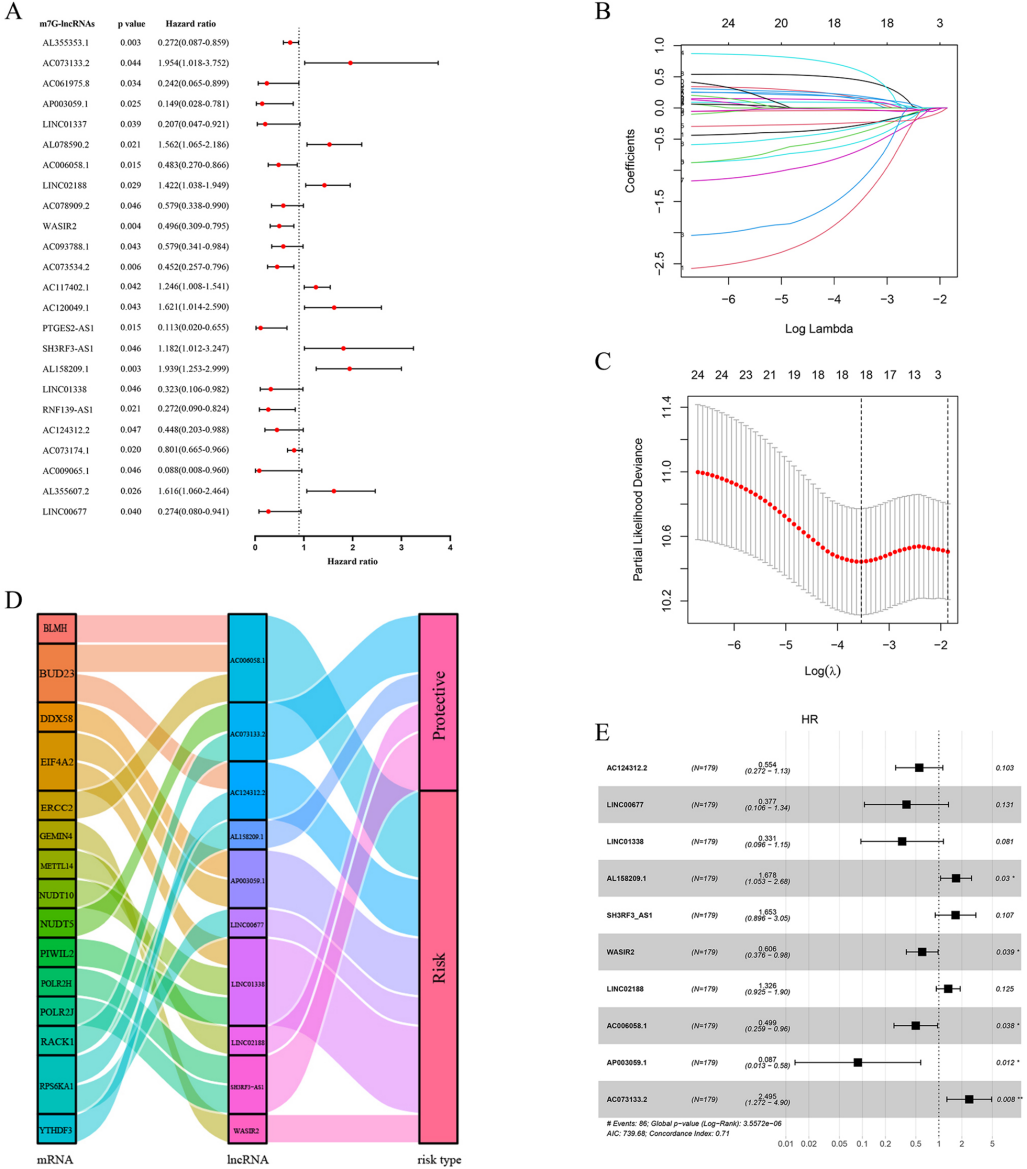
Establishment of the m7G prognostic model based on the m7G-related lncRNAs. **A** Forest plot of 24 m7G-related lncRNAs significantly associated with the OS (p < 0.05) by univariate Cox regression. **B** The LASSO coefficient distribution of 10 m7G-related lncRNAs. **C** The tenfold cross-validation for variable selection in the LASSO algorithm. **D** The Sankey diagram displays the detailed connections between the m7G regulators mRNA expression and the 10 m7G-related lncRNAs. **E** Forest plot shows the HR values of the 10 m7G-related lncRNAs with the OS by multivariate Cox regression

We randomly divided all BCa patients from the TCGA database into the training and test sets. The clinicopathologic features of these patients are presented in Table 1. Through univariate Cox regression analysis, we identified 24 lncRNAs significantly associated with the OS of BCa patients (p < 0.05) among 508 differentially expressed m7G-related lncRNAs (Fig. 3A). To reduce the possibility of overfitting, LASSO regression analysis was performed in the training set to screen further ten optimal m7G-related lncRNAs significantly correlated with the OS to establish the m7G prognostic model (Fig. 3B, C); four of which were protective factors, while another six lncRNAs tend to be risk factors (Fig. 3D). In order to better benefit the clinical application, we performed multivariate Cox regression analysis to determine regression coefficients of these ten lncRNAs (Table 2), transforming their expression values into a risk score. According to expression values and coefficients of these ten lncRNAs, the risk score of each BCa patient was determined with the equation below: Risk Score = (−0.5909 * AC124312.2) + (−0.9747 * LINC00677) + (−1.1046 * LINC01338) + (0.5176 * AL158209.1) + (0.5025 * SH3RF3-AS1) + (−0.5016 * WASIR2) + (0.2819 * LINC02188) + (−0.69546 * AC006058.1) + (−2.43718 * AP003059.1) + (0.9144 * AC073133.2).

**Table 1:** Clinicopathologic features of 358 BCa patients from the TCGA database

Variables	TCGA set (n = 358)	Training set (n = 179)	Test set (n = 179)
Age			
< 65	129	67	62
≥ 65	229	112	117
Gender			
Female	93	51	42
Male	265	128	137
Stage			
I	2	1	1
II	98	51	47
III	135	69	66
IV	123	58	65
T stage			
T0	1	0	1
T1	3	2	1
T2	111	55	56
T3	190	96	64
T4	53	26	27
N stage			
N0	238	122	116
N1	42	23	19
N2	72	32	50
N3	6	2	4
M stage			
M0	172	83	89
M1	186	96	90

**Table 2:** The detailed information of 10 lncRNAs in the m7G prognostic model

Gene	Ensemble ID	Location	HR	P value
AC124312.2	ENSG00000257647	chr15:25,027,736–25,032,047	0.448	0.0465
LINC00677	ENSG00000259717	chr14:103,120,847–103,123,007	0.274	0.0398
LINC01338	ENSG00000281327	chr5:82,807,475–82,860,008	0.323	0.0463
AL158209.1	ENSG00000230109	chr10:21,340,233–21,372,950	1.939	0.0029
SH3RF3-AS1	ENSG00000259863	chr2:109,127,327–109,128,930	1.812	0.0456
WASIR2	ENSG00000231439	chr16:22,910–25,123	0.496	0.0036
LINC02188	ENSG00000261175	chr16:86,710,122–86,742,083	1.422	0.0285
AC006058.1	ENSG00000261786	chr3:44,117,299–44,122,365	0.483	0.0145
AP003059.1	ENSG00000254731	chr11:86,703,099–86,714,092	0.147	0.0245
AC073133.2	ENSG00000228569	chr7:156,225,217–156,228,527	1.954	0.0442

**Fig. 4:**
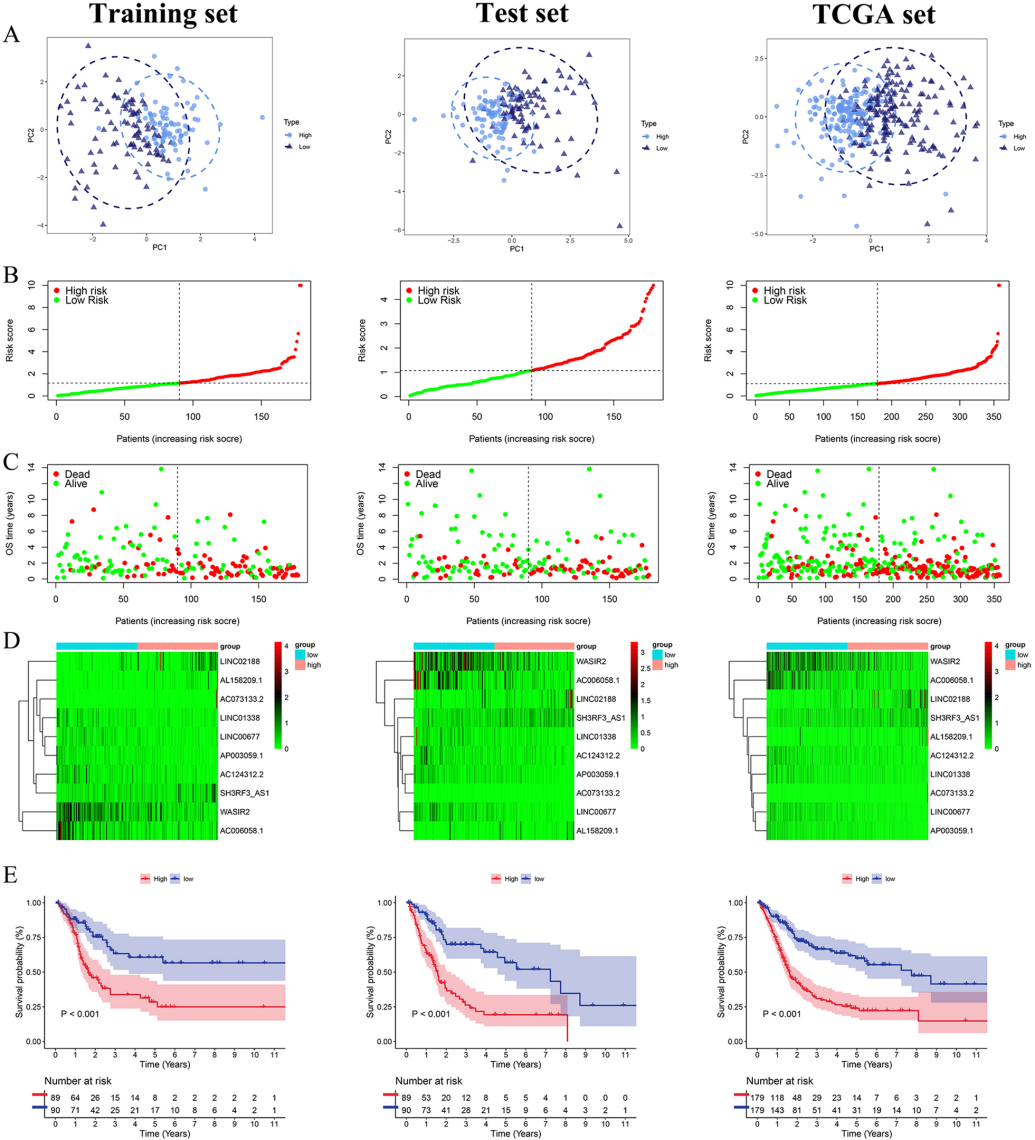
Validation of the m7G prognostic model of the 10 m7G-related lncRNAs. **A-C** Principal component analysis **(A)**, the risk score distribution of BCa patients **(B)**, and survival status distribution of BCa patients **(C)** for the training, test, and TCGA set, respectively. **D** The heatmaps of the 10 m7G-related lncRNAs expression between the high- and low-risk groups in the training, test, and TCGA sets. **E** Kaplan–Meier survival curves of the OS between the high- and low-risk groups in the training, test, and TCGA sets, respectively

**Fig. 5:**
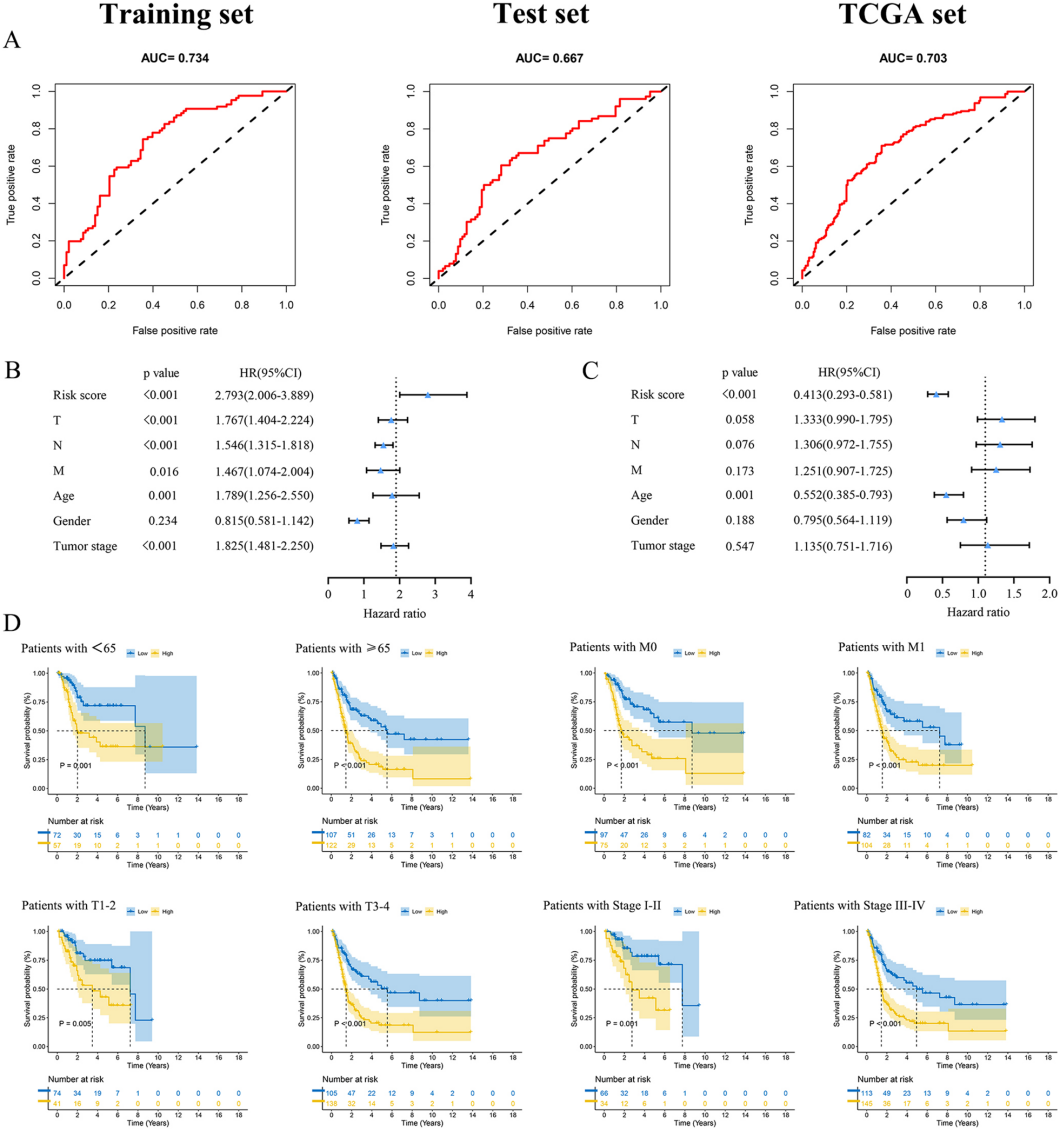
Independent prognostic factors and K-M survival curves of the OS stratified by different clinicopathologic features between the high- and low-risk groups in the TCGA set. **A** ROC analysis of the m7G prognostic model in the training, test, and TCGA sets, respectively. **B-C** Forest plot of the univariate Cox regression analysis **(B)**, and the multivariate Cox regression analysis **(C)** of the risk score and clinicopathologic features with the OS in the TCGA set. **D** K-M survival analysis of the OS stratified by age (< 65 or ≥ 65), M stage (M0 or M1), T stage (T1-2 or T3-4), and tumor stage (I-II or III-IV)

The multivariate Cox regression analysis revealed the mutual independence of the predictive capacity of these ten lncRNAs in the m7G prognostic model (Fig. 3E). According to the cut-off point of the median risk score, patients in the training, test, and TCGA sets were separated into the low-risk and high-risk groups. The differences in PCA, risk score distribution, and survival status between the low- and high-risk groups in these three sets revealed that the m7G prognostic model was reasonable in discriminating BCa patients (Fig. 4A–C). Compared to the low-risk group, patients in the high-risk group had significantly worse clinical outcomes (shorter OS, p < 0.001), as displayed in Fig. 4D. The heatmaps of the expression profiles of 10 prognosis-related lncRNAs showed that AC124312.2, LINC00677, LINC01338, WASIR2, AC006058.1, and AP003059.1 were negatively associated with the risk score, while AL158209.1, SH3RF3-AS1, LINC02188, and AC073133.2 were positively associated with the risk score (Fig. 4E). Analyses of ROC curves suggested that the m7G prognostic model exhibited good sensitivity and better effectiveness in predicting OS for BCa patients, with the areas under the ROC curve (AUC) 0.734 of the training set, 0.667 of the test set, and 0.703 of the TCGA set (Fig. 5A).

### Clinical significance of the m7G prognostic model

We conducted Cox regression analysis to determine whether the risk score calculated by our model was an independent prognostic risk factor affecting OS in BCa patients among clinicopathologic features (age, gender, TNM stage, and tumor stage). Univariate Cox regression analysis suggested that age, TNM stage, tumor stage, and risk score were significantly correlated with the OS (Fig. 5B). Based on the multivariate Cox regression analysis, it was confirmed that the risk score (HR = 0.413, p < 0.001) and age (HR = 0.552, p = 0.001) were independent prognostic factors in forecasting OS for BCa patients (Fig. 5C).

To assess the clinical significance of the m7G prognostic model, we conducted K-M survival analysis to determine whether this model retained an excellent predictive capacity of OS when patients were divided into different subgroups based on variable clinicopathologic features. For different subgroups stratified by clinicopathologic features, BCa patients in the high-risk group still had significantly lower OS (Fig. 5D, Additional file 6: Figure S1). Consequently, it was demonstrated that our model had broad clinical applicability in predicting OS for BCa patients regardless of variable clinicopathological features.

### Creation and assessment of nomogram

**Fig. 6:**
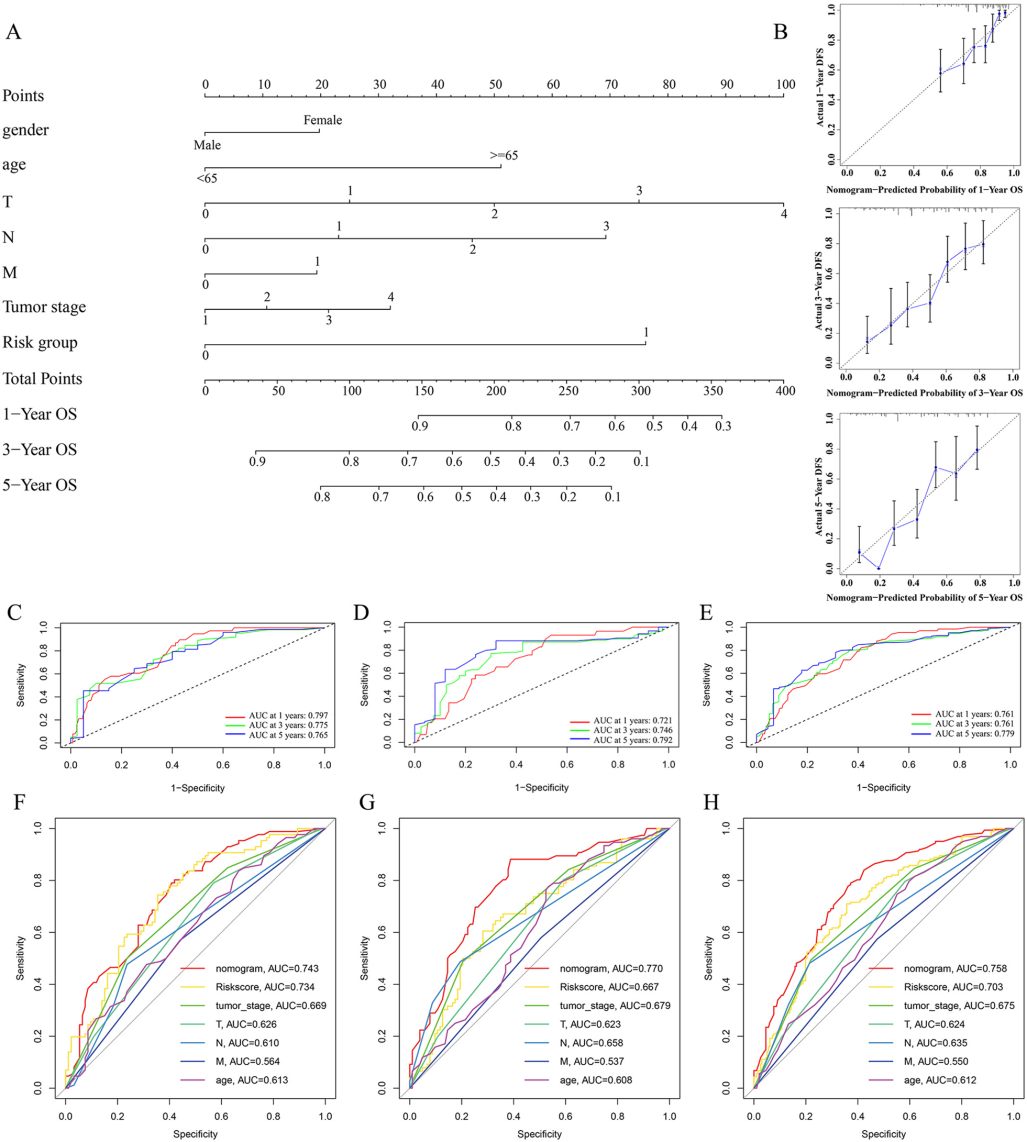
Construction and assessment of nomogram. **A** A nomogram integrated the risk score and clinical parameters.** B** The calibration curves displayed good uniformity between the predicted 1, 3, 5-years OS by the nomogram and the actual OS. **C-E** ROC curves analysis of the nomogram for 1, 3, and 5-years OS rate in the training **(C)**, test **(D)**, and TCGA sets **(E)**, respectively. **F–H** Multivariate ROC curves analysis for 5-years OS rate of the nomogram, risk score, and clinical parameters in the training **(F)**, test **(G)**, and TCGA sets **(H)**, respectively

To improve the predictive accuracy of 1, 3, and 5-years OS rates for BCa patients, we integrated clinicopathologic features and the risk score to create a nomogram (Fig. 6A). The calibration curves displayed good uniformity between the predicted 1, 3, 5-years OS rate by the nomogram and the actual OS rate (Fig. 6B). The C-index was 0.726 (95%CI 0.6938–0.7617, p = 0.006). According to time-dependent ROC analysis, the nomogram also exhibited excellent predictive performance in the training set, with the AUCs of 0.797, 0.775, and 0.765 for 1-, 3- and 5-years OS, respectively (Fig. 6C). The test set had 0.721, 0.746, and 0.792 AUCs (Fig. 6D). In the TCGA set, the AUCs were 0.761, 0.761, and 0.779 (Fig. 6E). The multivariate ROC curve of the nomogram, risk score and clinical features displayed that the AUC of the risk score was 0.734, significantly superior to clinical features (age, gender, tumor stage, and TNM stage) and just lower than the AUC of the nomogram in the training set (Fig. 6F). In the test and TCGA sets, we also performed the multivariate ROC curve analysis and found the same results with the training set (Fig. 6G, H). Based on the preceding, we concluded that the m7G prognostic model had an excellent predictive capacity of OS for BCa patients, and the nomogram could further improve the predictive accuracy.

### Functional enrichment analysis and gene set enrichment analysis

**Fig. 7:**
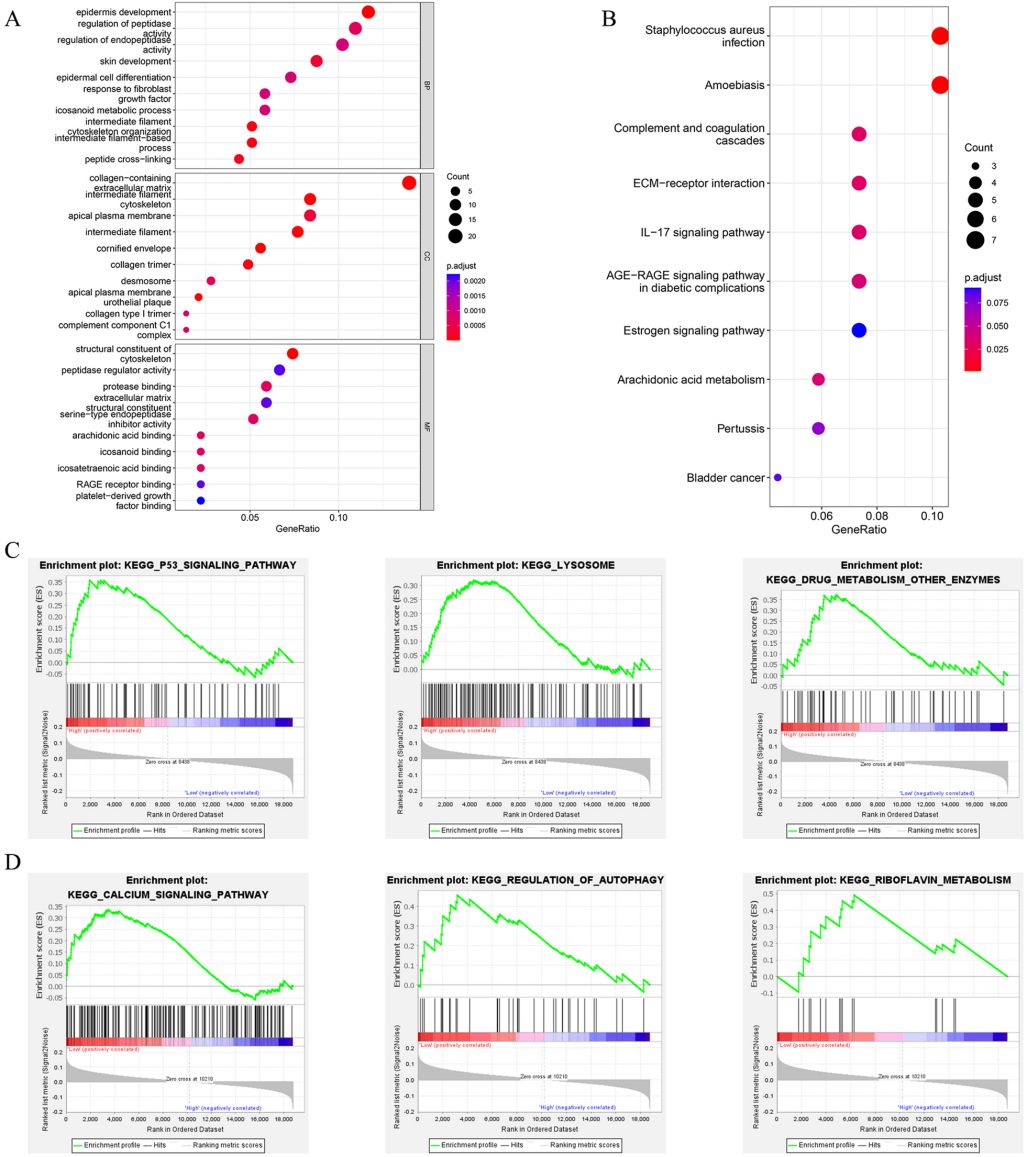
Functional enrichment analysis of the differentially expressed genes between the two groups. **A** GO enrichment of 146 DEGs.** B** KEGG enrichment of DEGs. **C-D** Pathways significantly enriched in the high-risk group **(C)** and low-risk group **(D)** in GSEA analysis

To further investigate the molecular mechanisms behind apparent discrepancies between the two groups, we performed GO and KEGG analysis based on the 146 differentially expressed genes (|log_2_FC|≥ 1 and p. adj. < 0.05) between the two groups Additional file 3: Table S3). GO enrichment analysis consists of three components: biological process (BP), cellular component (CC), and molecular function (MF). As to BP, DEGs are mainly enriched in epidermis development, regulation of peptidase activity, and regulation of endopeptidase activity. As to MF, DEGs are mainly enriched in structural constituent of cytoskeleton, peptidase regulator activity, and protease binding (Fig. 7A). Then, the KEGG analysis indicated that these DEGs were mainly enriched in bladder cancer, complement and coagulation cascades, ECM-receptor interaction, arachidonic acid pathway, and IL-17 signaling pathway (Fig. 7B). Furthermore, the GSEA of the KEGG pathway (Additional file 4: Table S4) revealed that the pathways of P53 signaling pathway, lysosome, and drug metabolism other zymes were mainly enriched in the high-risk group (Fig. 7C), while calcium signaling pathway, regulation of autophagy, and riboflavin metabolism were mainly enriched in the low-risk group (Fig. 7D).

### Analysis of tumor immune microenvironment

**Fig. 8:**
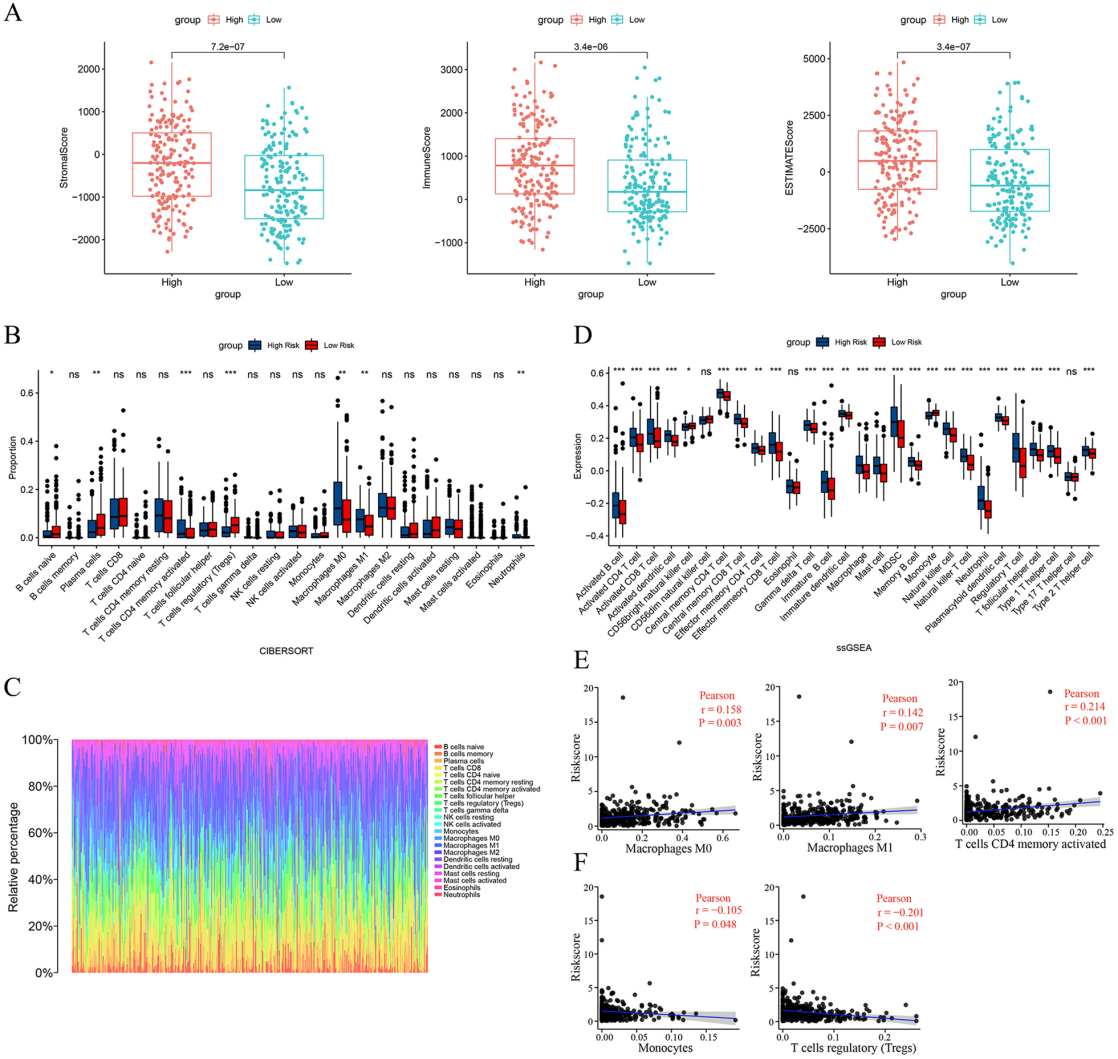
Relationship between the m7G prognostic model and tumor immune microenvironment. **A** Comparison of immune-related scores between the high- and low-risk groups. **B-C** Differences of immune cell infiltration levels evaluated by CIBERSORT algorithm. **D** ssGSEA analysis of the immune cell infiltration between the two groups. **E–F** The correlation of the risk score and immune cell scores calculated by CIBERSORT algorithm. *P < 0.05, **P < 0.01, ***P < 0.001, ns, no significance

Due to the significance of TME in tumorigenesis, progression and treatment, we investigated the correlations between the immunological signature and the m7G prognostic model. Firstly, we performed the ESTIMATE algorithm to calculate the immune, stromal, and estimate scores of each BCa patient in the TCGA set (Additional file 5: Table S5). The results showed that patients in the high-risk group had considerably higher immune, stromal, and estimate scores than in the low-risk group (Fig. 8A), revealing significant differences in TME between the two groups. Next, we applied the CIBERSORT algorithm to evaluate the proportion of 22 immune cells. It was found that the high-risk group was infiltrated by a higher portion of T cells CD4 memory activated, Macrophages M0, Macrophages M1, and Neutrophils, while a lower portion of B cells naive, Plasma cells, and T cells regulated (Tregs) than the low-risk group (Fig. 8B, C). According to ssGSEA algorithm, we found that the high-risk group had higher infiltration levels of B cell, CD4 + T cell, CD8 + T cell, dendritic cell, immature B cell, immature dendritic cell, macrophages, mast cell, MDSC, NK cell, neutrophil, plasmacytoid dendritic cell, regulatory T cell, T follicular helper cell, T helper cell. In contrast, CD56 NK cell and monocyte were lower infiltrated in the high-risk group (Fig. 8D). We also explored the relationship between the risk score and infiltration values of some representative immune cells using Pearson correlation analysis. We found that Macrophages M0, Macrophages M1, and T cell CD4 + memory were positively correlated with the risk score (Fig. 8E), while monocyte and Tregs were negatively correlated with the risk score (Fig. 8F).

### Prediction of the sensitivity to antitumor drug with the risk score

**Fig. 9:**
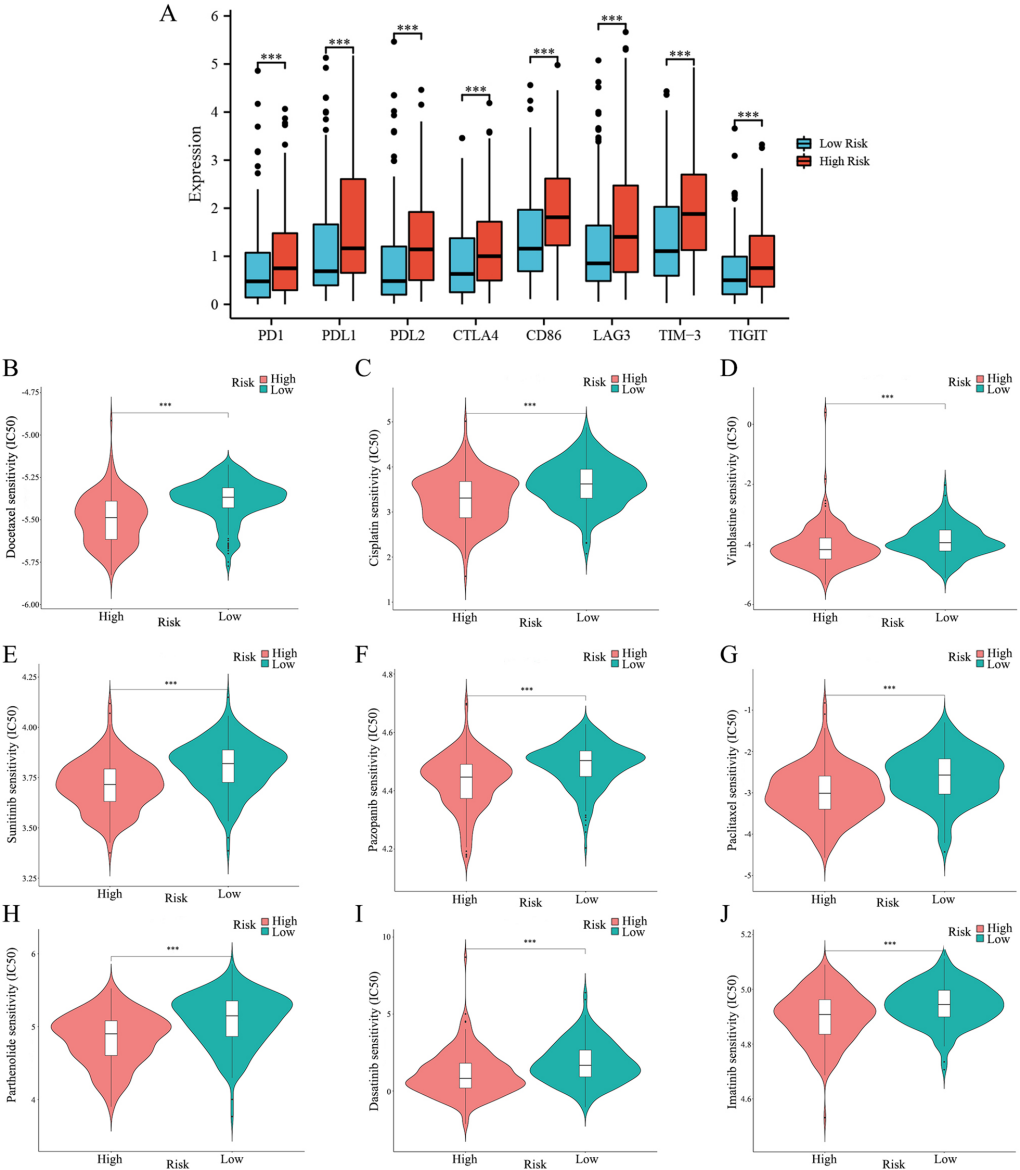
Immune checkpoint genes expression and prediction of sensitivity to chemotherapeutic drugs. **A** Differentially expressed immune checkpoint genes between the two groups. **B-J** Chemotherapeutic drugs with low IC50 in the high-risk group. *P < 0.05, **P < 0.01, ***P < 0.001

**Fig. 10:**
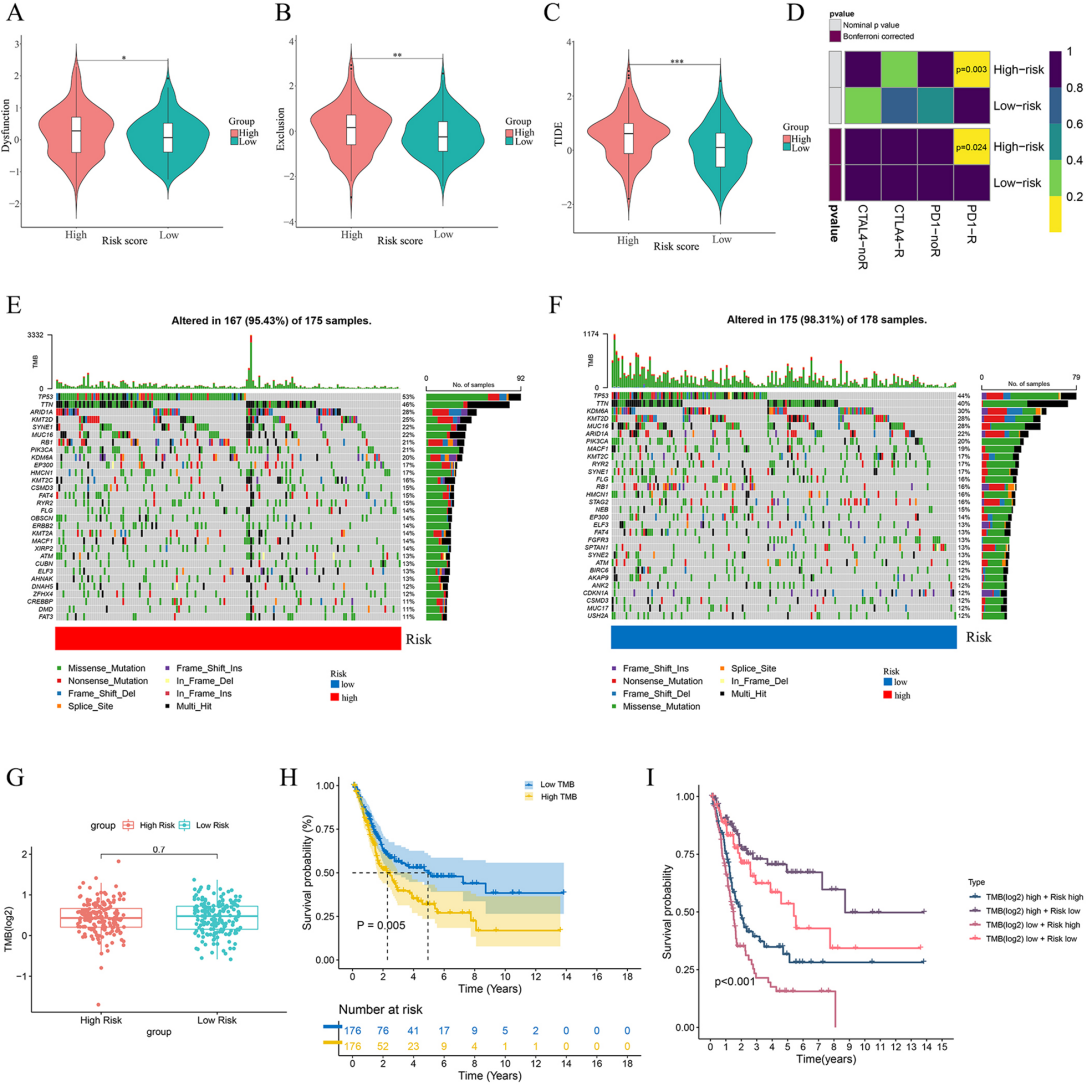
Analysis of tumor mutation burden (TMB). **A-C** Distribution of the dysfunction scores **(A)**, exclusion scores **(B)**, and TIDE scores **(C)** between the two groups.** D** Prediction of the response to anti-PD1 and anti-CTLA4 immunotherapy in the high- and low-risk groups by the submap algorithm. **E–F** Waterfall plots of mutation characteristics in the two groups. **G** Differences of TMB between the two groups. **H** K-M survival curve of the OS between the high- and low-TMB groups.** I** Kaplan–Meier survival curve of the OS stratified by both TMB and risk score. *P < 0.05, **P < 0.01, ***P < 0.001, ns: no significance

We compared the expression levels of critical immune checkpoint genes between the two groups to explore the potential benefits of the m7G prognostic model in guiding immunotherapy. As shown in Fig. 9A, patients in the high-risk group had higher expression levels of PD-1, PD-L1, PD-L2, CTLA-4, CD86, LAG3, TIM-3, and TIGIT. Then, we assessed the responses of the two groups to chemotherapeutic drugs by the half-maximal inhibitory concentration (IC50) values. The results showed that BCa patients in the high-risk group were more susceptible to docetaxel, cisplatin, vinblastine, sunitinib, pazopanib, paclitaxel, parthenolide, dasatinib, and imatinib (Fig. 9B–J), whereas patients in the low-risk group were more susceptible to methotrexate, nilotinib and metformin (Additional file 6: Figure S2). Besides, we evaluated the efficacy of immune checkpoint inhibitors (ICIs) between the two groups using the TIDE algorithm. Compared to the low-risk group, the high-risk group presented significantly higher TIDE scores and T cell exclusion scores (Fig. 10A–C). We also found that patients in the high-risk group might benefit more from anti-PD-1 treatment (Fig. 10D, Bonferroni corrected p = 0.003). Neoadjuvant cisplatin-based chemotherapy (cisplatin, docetaxel, vinblastine, and methotrexate) has been standard for two decades in advanced BCa patients [34]. In summary, BCa patients with high-risk scores may be more susceptible to neoadjuvant cisplatin-based chemotherapy and anti-PD-1 immunotherapy.

### TMB analysis

We obtained the somatic mutation data of BCa patients from the TCGA database and analyzed the genetic alterations between the two groups. It was found that TP53 and TTN were the top 2 most mutated genes in the two groups, and patients in the high-risk groups had higher mutation rates of TP53 than those in the low-risk group (Fig. 10E, F). However, neither group appeared to have significant differences in TMB generally (Fig. 10G). K-M survival analysis revealed that patients in the high-TMB group had significantly worse OS than those in the low-TMB group (Fig. 10H). Then, we combined the risk score and TMB to predict the prognosis of BCa patients. It was noted that patients in the high-risk + low TMB group had the worst outcomes, while those in the low-risk + high TMB group had the best outcomes (Fig. 10I).

### Expression level 10 m7G-related lncRNAs of in bladder cancer cell lines

**Fig. 11:**
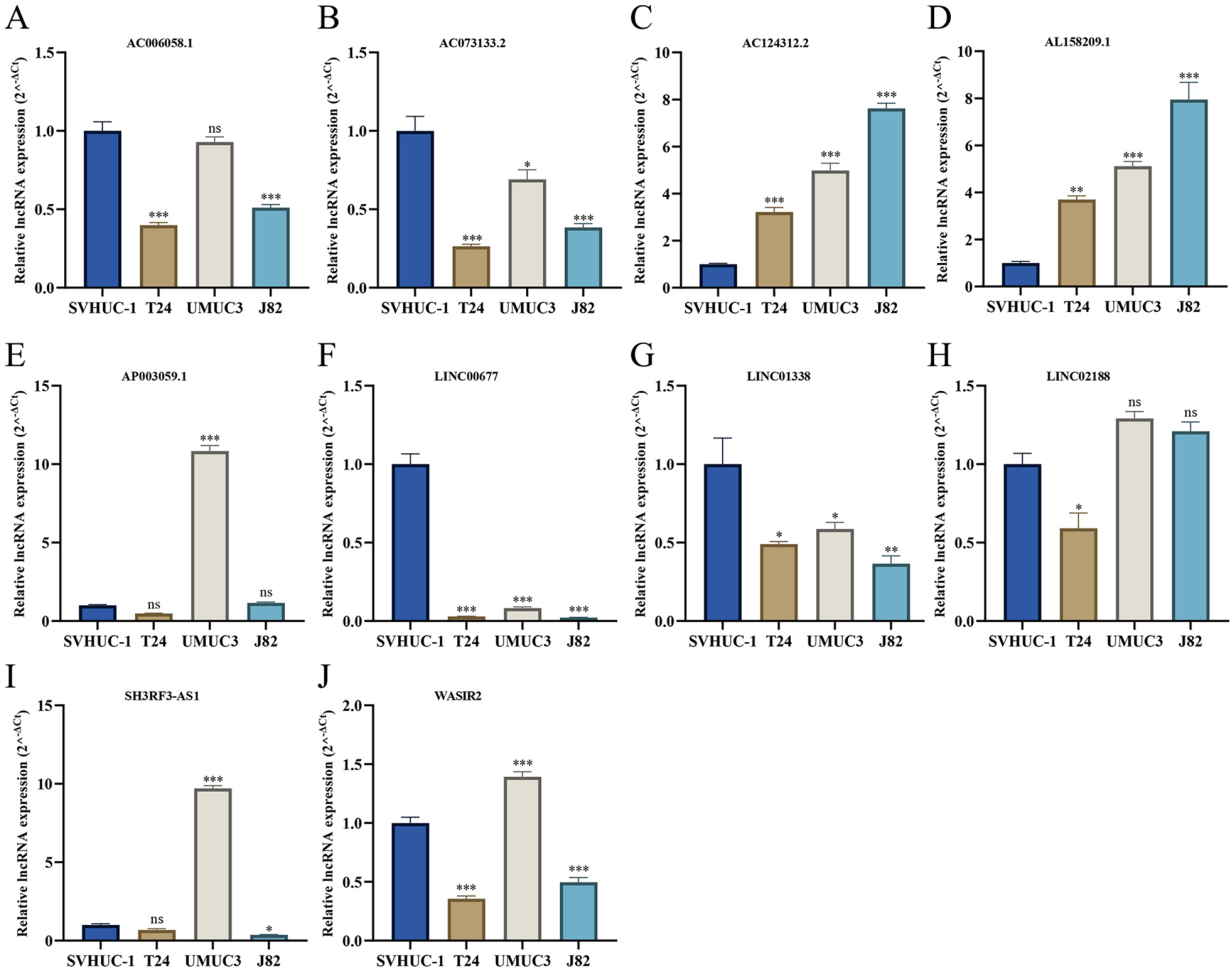
The qRT-PCR results of 10 m7G-related lncRNAs in three BCa cell lines. **A** qRT-PCR result of AC006058.1. **B** qRT-PCR result of AC073133.2. **C** qRT-PCR result of AC124312.2. **D** qRT-PCR result of AL158209.1. **E** qRT-PCR result of AP003059.1.** F** qRT-PCR result of LINC00677. **G** qRT-PCR result of LINC01338. **H** qRT-PCR result of LINC02188. **I** qRT-PCR result of SH3RF3-AS1.** J** qRT-PCR result of WASIR2. *P < 0.05, **P < 0.01, ***P < 0.001, *ns* no significance

We further validated the expression levels of 10 m7G-related lncRNAs in BCa cells lines T24, UMUC3, and J82 by qRT-PCR. As shown in Fig. 11, we found that AC006058.1, AC073133.2, LINC00677, and LINC01338 were significantly downregulated in BCa cell lines compared with those in the SVHUC-1 cells. Meanwhile, the expression levels of AC124312.2 and AL158209.1 were significantly upregulated in BCa cell lines compared with those in the SVHUC-1 cells. We also observed that AP003059.1 and SH3RF3-AS1 were only upregulated in UMUC3 cell lines, and LINC02188 was downregulated in T24 cell lines. However, in our bioinformatics analyses, AC073133.2 and LINC02188 were significantly upregulated in the BCa samples, and AC124312.2 and AP003059.1 were significantly downregulated in the BCa samples, which were inconsistent with the results of qPCR. As for lncRNA WASIR2, we found that it was significantly upregulated in the UMUC3 BCa cell lines and showed significantly downregulated level in the T24 and J82 BCa cell lines.

## Discussion

Among the most common malignancies, BCa is characterized by high heterogeneity, leading to miscellaneous clinical outcomes. Over the last few decades, the advent of immunotherapy has remodeled the treatment paradigm and accelerated the development of individualized precision therapy for cancers. The immunotherapy, such as PD-1, PD-L1, and CTLA-4 inhibitors, has improved the prognosis of BCa patients but is still in the initial stage, and more clinical trials in multi-center are urgently needed [5, 35]. The existing body of studies on m7G suggested that its abnormal expression would heighten the translation of oncogenic mRNAs and boost tumorigenesis, advancement, and metastasis in various cancers, especially in BCa [13–15, 36].

We gained 20 differentially expressed m7G-related genes between normal samples and BCa samples, including METTL1 and WDR4. Previous studies have demonstrated that METTL1-mediated m7G tRNA modifications can promote the development of BCa [12]. We concluded that m7G modifications of other RNA types might significantly influence the carcinogenesis and advancement of BCa. Subsequently, the functional analyses demonstrated that these 20 m7G-related genes were mainly enriched in the activity of tRNA (guanine)-methyltransferase, transcription regulator complex, and RNA polymerase II transcription regulator complex, which were involved in the process of m7G modifications [9, 37, 38]. In addition, these genes were also enriched in MAPK signaling pathway, ErbB signaling pathway, mTOR signaling pathway, and Wnt signaling pathway, which play a key role in tumor initiation, progression, and invasion. These results revealed that m7G modification might relate to the above pathways.

In our study, we collected 358 BCa samples and 18 normal tissues with raw RNA-seq transcriptome data and complete clinical information from the TCGA database. We separated all BCa patients into the training and test sets at random in order to evaluate and quantify the significance of m7G-related lncRNAs for BCa patients. Then, we developed an innovative m7G prognostic model (risk score) composed of ten optimal m7G-related lncRNAs significantly associated with the OS in the training set. Among these lncRNAs, AC124312.2, LINC00677, LINC01338, WASIR2, AC006058.1, and AP003059.1 were risk factors for BCa, whereas AL158209.1, SH3RF3-AS1, LINC02188, and AC073133.2 tended to be protective factors. It was found that lncRNA WASIR2 was upregulated in colon cancer tissue and had independent prognostic significance for stage II colon cancer [39]. Li et al. discovered that lncRNA WASIR2 might be a tumor suppressor gene regulated by DNA methylation and could act as an independent prognostic factor for lung adenocarcinoma [40]. In addition, Zhou et al. identified WASIR2 as a B-cell-specific lncRNA that was highly expressed in B cells compared to BCa cells [41]. Furthermore, they developed a prognostic lncRNA signature comprising WASIR2, validated as an independent prognosis indicator for BCa patients. The result of qPCR in our study revealed that lncRNA WASIR2 was significantly downregulated in the T24 and J82 BCa cell lines, consistent with our bioinformatic analysis. These findings substantiated our study, which proposed that lncRNA WASIR2 may serve as a tumor suppressor gene, inhibiting the development of BCa. For the other nine lncRNAs, no relevant studies and expression levels in tumors were reported. We further validated their expression levels in BCa cell lines by qRT-PCR and found that AC006058.1, AC073133.2, LINC00677, and LINC01338 were significantly downregulated in BCa cell lines. The expression levels of AC124312.2 and AL158209.1 were significantly upregulated in BCa cell lines. Moreover, in our bioinformatics analyses, AC073133.2 and LINC02188 were significantly upregulated in the BCa samples, and AC124312.2 and AP003059.1 were significantly downregulated in the BCa samples, which were inconsistent with the results of qPCR. In view of the inconsistent results from qPCR and bioinformatics analyses, we surmised that the discrepancy might be due to the following factors: firstly, normal samples in our study from the TCGA database only contained eighteen, and BCa tissues (including NMIBC and MIBC) may show heterogeneity; secondly, different transcripts of lncRNA are generated during the RNA production, and the TCGA database may only contains a part of some lncRNA transcripts; finally, selection bias is inevitable that may affect the precision and accuracy of our bioinformatics analyses, and qPCR in BCa cell lines is essential to verify the expression levels of these lncRNAs.

We determined each patient’s risk scores in line with the m7G prognostic model built from the training set and then split all of the patients into the high-risk and low-risk groups based on the median value of risk scores. In the TCGA, training, and test sets, PCA and risk score distribution demonstrated that our model was good at discriminating BCa patients. The survival curves showed that the high-risk group patients had significantly shorter OS than the low-risk group patients. The ROC curve analyses further validated the better effectiveness of our model in predicting OS for BCa patients compared with other clinicopathologic features, indicating that our prognostic model is an accurate predictor of the prognosis for BCa patients. We verified that the risk score was an independent OS prognostic indicator for BCa patients. Regardless of different clinicopathological variables, our prognostic model still showed an excellent independent predictive power of the OS. Then, we integrated clinicopathological features and the risk score to create a nomogram. The calibration curves and time-dependent ROC curves revealed that the nomogram further improves the m7G prognostic model’s predictive accuracy of the OS for BCa patients.

The KEGG results revealed that the DEGs between the low- and high-risk groups were mainly enriched in bladder cancer, complement and coagulation cascades, ECM-receptor interaction, arachidonic acid pathway, and IL-17 signaling pathway. As an important metabolic pathway, arachidonic acid pathway plays a vital role in the initiation and development of many cancers. The natural products that inhibit this pathway may prevent cancer initiation and become a novel tumor treatment strategy [42]. Thus, these illustrated that m7G-related lncRNAs were significantly related to BCa and may involve in the development, invasion, and metastasis of BCa via arachidonic acid pathway, suggesting that these lncRNAs may be novel therapeutic targets for BCa. According to GSEA, P53 signaling pathway and lysosome were significantly enriched in the high-risk group, while the calcium signaling pathway and regulation of autophagy were primarily enriched in the low-risk group. These enriched pathways were all significantly related to cancer, suggesting that these pathways were associated with m7G-related lncRNAs. P53 signaling pathway had critical roles in cancer biology and oncology as a tumor suppressor pathway, and inactivation or mutation of the TP53 gene could promote the proliferation, invasion, and metastasis in most tumors, including BCa [43, 44]. Additionally, calcium signaling pathway was involved in the development of tumors and regulated the tumor microenvironment (TME) through recruiting and remodeling its toolkit [45]. The primary and metastatic TME are both essential drivers of tumor proliferation and progression through a potential way of calcium signaling pathway [46]. Calcium signaling pathway is highly dependent on the activity of the calcium channels that regulate the intracellular concentration of Ca^2+^. The intracellular concentration of Ca^2+^ plays an important role in cancer cell death and proliferation, and an increased level of it is necessary for the efficiency and function of cytotoxic T lymphocytes (CTL) and NK cell in targeting cancer cell death [47].

TME, which comprises fibroblasts, innate immune cells, adaptive immune cells, stromal cells, and endothelial cells, has attracted much attention from research and clinical trials, being seen as a potential therapeutic target in the innovation and advancement of cancer treatments strategy [48, 49]. The high-risk group had significantly higher estimate, stromal, and immune scores, which represented a more complicated TME and might be one of the reasons for the patients’ poorer outcomes in this group, which were in accordance with previous studies [50]. In addition, the findings of ssGSEA revealed that the high-risk group showed higher infiltration levels of macrophages, dendritic cell, mast cell, regulatory T cell, CD4 + T cell, and CD8 + T cell. We also found that the infiltration of T cell CD4 + memory, Macrophages M1, and Macrophages M0 was positively correlated with the risk score. Macrophages generally play a protumoral role and enhance cancer cell invasion and migration. They also showed immunosuppressive function to help cancer cells escape attack from CD8 + T cells and natural killer cells, and high infiltration of macrophages in TME was associated with poor prognosis of cancer patients [51]. Substantial evidence revealed that a higher proportion of tumor-associated macrophages was associated with a high rate of lymphatic metastasis and indicated poor prognosis of BCa patients [24, 52]. Furthermore, it was found that BCa cells would recruit more CD4 + T cells to its surrounding, which could promote the proliferation and invasion of BCa cells [53]. Several studies have demonstrated increased CD8 + T cell infiltration is related to poor OS and recurrence-free survival in BCa [54]. These results above may account for the worse prognosis of the high-risk group patients. Studies over the past decades have indicated that the high immune cells infiltration state showed a propensity towards more sensitive immunotherapeutic effectiveness, and more T-cell infiltration was significantly related to more efficacy of anti-PD1/PD-L1 treatments [49, 55]. According to previous studies, BCa patients with high levels of PD-L1 expression are inclined to have poor clinical outcomes; however, the high PD-L1 expression indicated more efficacy in the anti-PD-L1 immunotherapy [49, 56]. It has been demonstrated that anti-PD1/PD-L1 treatments, including atezolizumab, nivolumab, avelumab, and pembrolizumab could prolong the survival time for cisplatin-ineligible BCa patients [57]. Our study found that the high-risk group patients consistently exhibited greater T-cell infiltration and higher expression levels of PD-1, PD-L1, TIGIT, PD-L2, CTLA-4, CD86, LAG3, and TIM-3 than the low-risk group patients. Then, to evaluate the response to immune checkpoint inhibitor (ICB) in BCa, we calculated TIDE scores of the two groups. It was observed that the high-risk group patients presented higher TIDE scores and responded to anti-PD-1 immunotherapy more sensitively. Due to the remarkable effectiveness of chemotherapy in advanced BCa patients [6, 57], we evaluated the susceptibility to common anti-BCa drugs. Interestingly, patients in the high-risk group showed more sensitivity to neoadjuvant cisplatin-based chemotherapy for advanced BCa, containing docetaxel, vinblastine, and cisplatin; they also showed higher sensitivity to sunitinib, paclitaxel, imatinib, parthenolide, dasatinib, and pazopanib. Conversely, the low-risk group patients were more sensitive to methotrexate and nilotinib. To sum up, the above findings suggested that BCa patients in the high-risk group are more suitable for neoadjuvant cisplatin-based chemotherapy and anti-PD-1 treatment, and the low-risk group patients are more sensitive to methotrexate and nilotinib. These results further demonstrated that the m7G prognostic model (risk score) could be a robust tool to assist clinicians in providing an individual-based treatment regime for BCa patients.

During the last few years, the in-depth mining of oncology databases, such as the TCGA database containing clinicopathological information and gene expression levels of cancer patients, has been flourishing and aided researchers in discovering tumor biomarkers and therapeutic targets. Many studies have emerged that mining information from oncology databases to construct lncRNA-based or gene-based signatures to predict the prognosis of cancer patients, including BCa. For instance, a novel cuproptosis-related lncRNA signature was identified to predict prognosis and immune landscape in BCa [58]. Lu et al. identified 12 pyroptosis-associated lncRNAs to construct the signature to predict prognosis and TME of BCa [59]. Due to the little known about the mechanisms and prognostic features of m7G-related lncRNAs in BCa, we identified ten m7G-related prognostic lncRNAs and constructed a prognostic model. In comparison to the BCa models cited above, our model is beneficial in that it provides comprehensive treatment strategies, including chemotherapy and immunotherapy, to ameliorate the outcomes for BCa patients; it has a higher AUC value (0.734) than the cuproptosis-related lncRNA signature (AUC = 0.692) and contains fewer lncRNAs than Lu’s model, improving the convenience for clinicians to apply our model to predict the prognosis of BCa patients.

However, we must recognize that our study had some limitations. Firstly, our analysis refers to a retrospective study of public data, and selection bias is inevitable, which may affect the precision and accuracy of our results. Secondly, external databases and prospective clinical studies are necessary to validate our prognostic model. Finally, the predictive power of our model in response to chemotherapy and anti-PD1 treatment needs to be validated in independent clinical trials.

## Conclusion

In conclusion, we performed a comprehensive bioinformatics and identified a novel m7G prognostic model (risk score), which can be applied to accurately predict the prognosis and ameliorate the outcomes for BCa patients. Moreover, our model is able to provide robust directions for clinicians to develop better individual-based and precise treatment strategies for BCa patients.

## Supplementary Information

**Additional file 1: Table S1**. 143 m7G-related genes.

**Additional file 2: Table S2**. Primers for qRT-PCR.

**Additional file 3: Table S3**. Differentially expressed genes between the low- and high-risk groups.

**Additional file 4: Table S4**. GSEA analysis of enriched pathways in the high- and low-risk groups.

**Additional file 5: Table S5**. The immune, stromal, and estimate scores of each BCa patient in the TCGA set.

**Additional file 6: Fig. S1**. K-M survival analysis of the OS stratified by genderand N stage.

**Additional file 7: Fig. S2**. Chemotherapeutic drugs with low IC50 in the low-risk group. *P < 0.05, **P < 0.01, ***P < 0.001.

## Data Availability

The datasets supporting the conclusions of this article are included within the article and its additional files. More detailed data is available from the corresponding author on reasonable request.

## Abbreviations

BCa: Bladder cancer
NMIBC: Non-muscle-invasive BCa
MIBC: Muscle-invasive BCa
m7G: N7-methylguanosine
TCGA database: The Cancer Genome Atlas database
TME: Tumor microenvironment
TMB: Tumor mutation burden
OS: Overall survival
GO: Gene ontology
BP: Biological process
CC: Cellular component
MF: Molecular function
KEGG: Kyoto encyclopedia of genes and genomes
GSEA: Gene set enrichment analysis
LASSO: The least absolute shrinkage and selection operator
K-M survival curve: Kaplan–Meier survival curve
ROC curve: Receiver operating characteristic curve
AUC: The area under the curve
PCA: Principal component analysis
DEGs: Differentially expressed genes
ssGSEA: Single-sample gene set enrichment analysis
IC50: The half-maximal inhibitory concentration
TIDE: The tumor immune dysfunction and exclusion
ICI: Immune checkpoint inhibitor
