# Supplementary figures and images for: An innovative model based on N7-methylguanosine-related lncRNAs for forecasting prognosis and tumor immune landscape in bladder cancer
Source: Cancer Cell Int. 2023 May 8;23:85. doi: 10.1186/s12935-023-02933-7 (PMC10165842; doi:10.1186/s12935-023-02933-7)

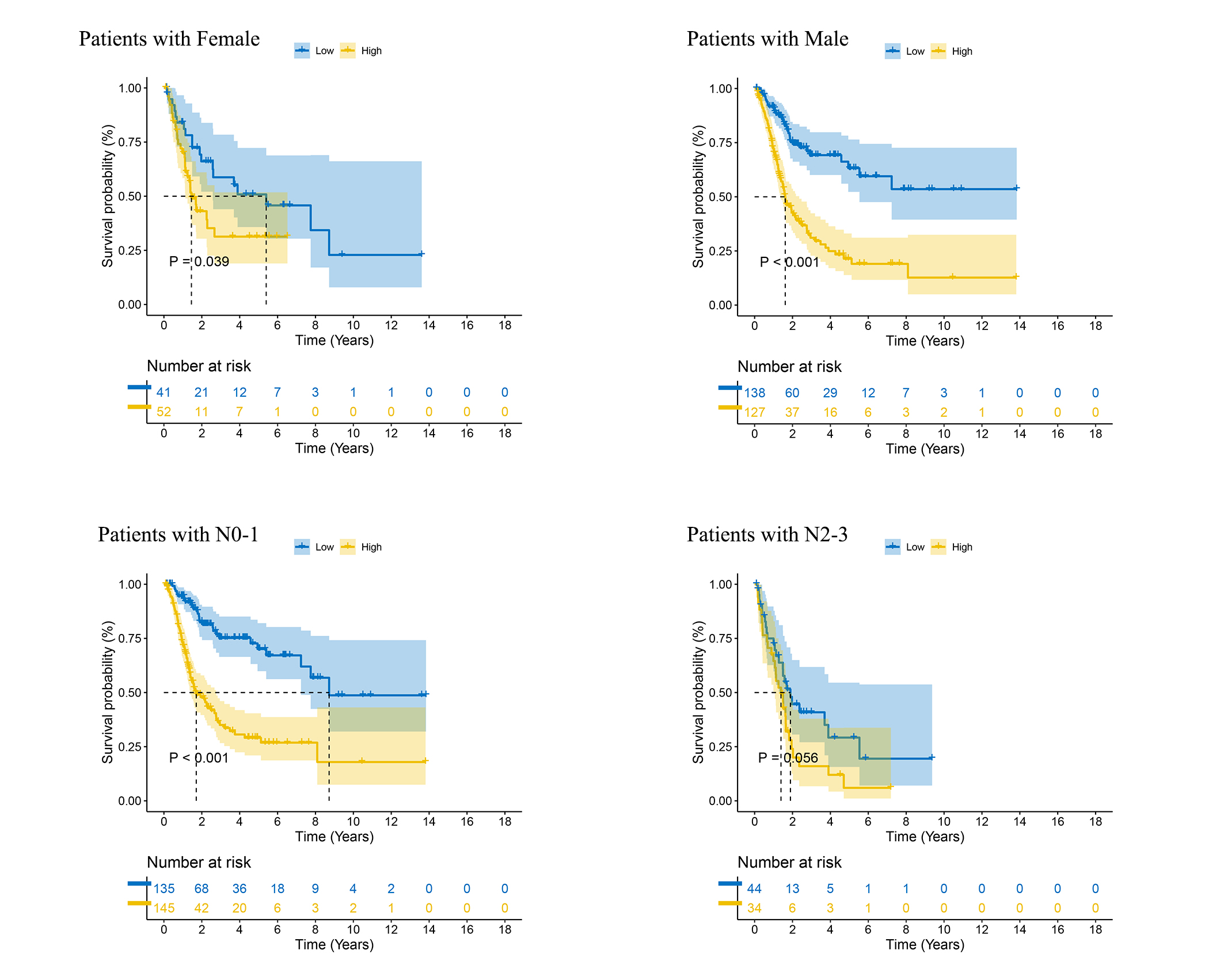

Supplement: Supplementary file 6 — Additional file 6: Fig. S1. K-M survival analysis of the OS stratified by genderand N stage. [file 12935_2023_2933_MOESM6_ESM.jpg]

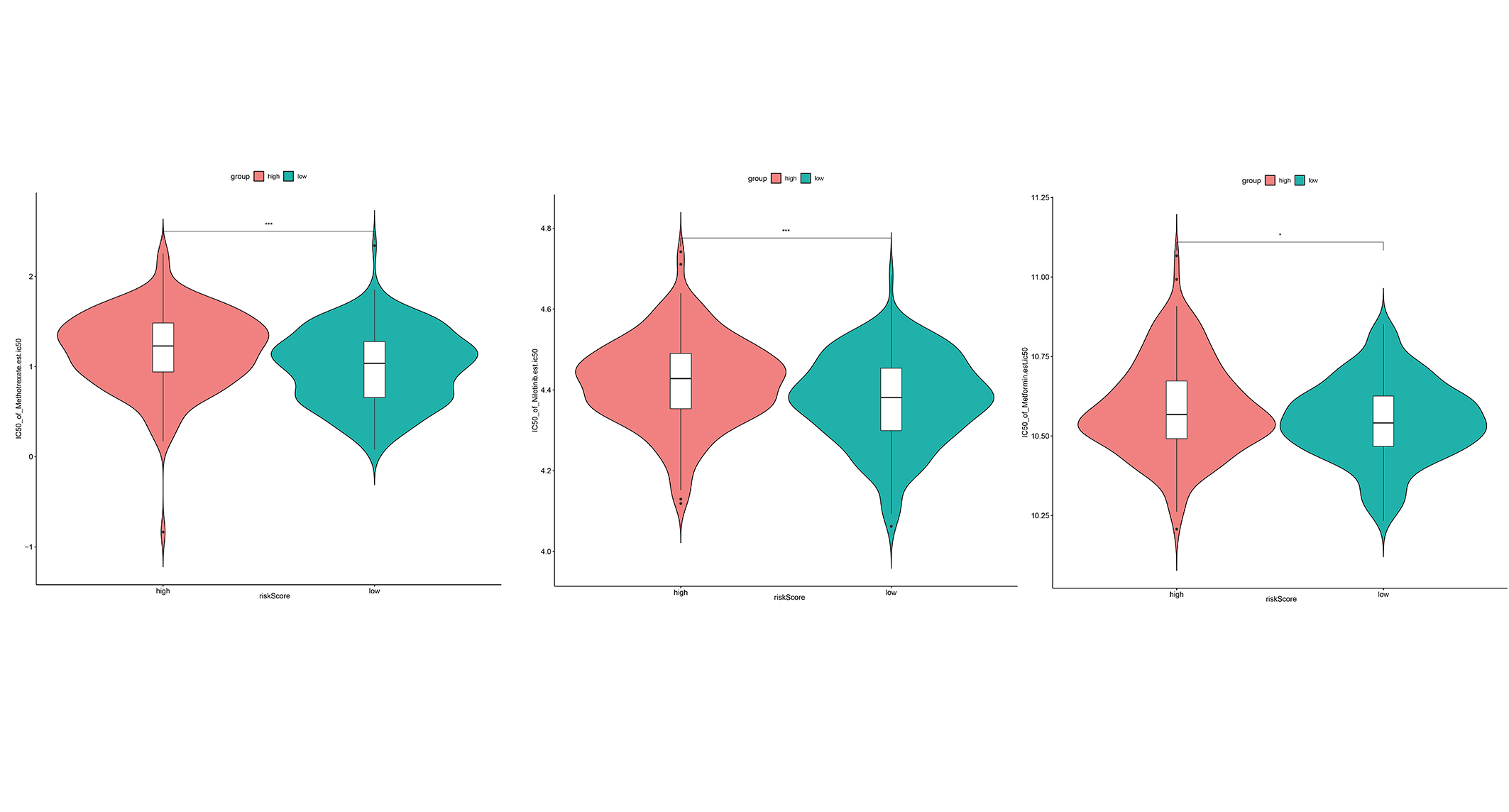

Supplement: Supplementary file 7 — Additional file 7: Fig. S2. Chemotherapeutic drugs with low IC50 in the low-risk group. *P < 0.05, **P < 0.01, ***P < 0.001. [file 12935_2023_2933_MOESM7_ESM.jpg]
